# Dynamic curvature topography for evaluating the anterior corneal surface change with Corvis ST

**DOI:** 10.1186/s12938-015-0036-2

**Published:** 2015-06-04

**Authors:** Chunhong Ji, Jinhua Yu, Tianjie Li, Lei Tian, Yifei Huang, Yuanyuan Wang, Yongping Zheng

**Affiliations:** Department of Electronic Engineering, Fudan University, Shanghai, 200433 China; Key Laboratory of Medical Imaging Computing and Computer Assisted Intervention of Shanghai, Shanghai, China; Interdisciplinary Division of Biomedical Engineering, Hong Kong Polytechnic, Hong Kong, China; Department of Ophthalmology, Chinese PLA General Hospital, Beijing, 100853 China

**Keywords:** Dynamic parameter, Segmentation, Cornea, Corvis ST

## Abstract

**Background:**

The measurement of dynamic parameters, such as the length of applanation and the amplitude of deformation, is significant for evaluating corneal properties. Most of the corneal properties (related to shape) including the anterior corneal curvature and the thickness of cornea can be easily measured using some existing techniques. However, they only provide the static or pseudo-dynamic analysis. Based on Corvis ST images, the dynamic features after corneal boundaries detection and parameter estimation will be helpful for corneal analysis.

**Material:**

The study included 40 eyes in normal group (ranging from 19 to 45 years old) and 30 eyes in keratoconus group (ranging from 16 to 40 years old). These eyes were examined by Corvis ST and for each one a sequence of 140 images was obtained. Besides, 11 subjects of each group were also tested by Pentacam.

**Methods:**

By analyzing the video from the Corvis ST imaging, the fully dynamic curvature topography is proposed to evaluate the response of the anterior corneal surface to the air puff. The new method not only quantitatively measures the intact variation of anterior corneal surface but also provides an intuitive way to observe the dynamic change of the anterior corneal surface in the whole air stream process. The proposed method consists of three main steps: cornea segmentation, curvature estimation and integrated visualization. An automatic segmentation method based on the combination of prior knowledge with phase symmetry and asymmetry theory is firstly presented to detect the corneal boundaries. The Landau-new method is then used to estimate the anterior corneal surface. The corneal dynamic topography is finally obtained by combining the dynamic parameters with the original Corvis ST video, which is an improvement of the fusion technique proposed by Li et al.

**Results and conclusion:**

By comparing the segmentation results with manual method and built-in method of Corvis ST, the accuracy and robustness of our proposed segmentation method is demonstrated. The correctness of the estimated corneal anterior curvatures is also evaluated by comparing it with that of Pentacam which is considered to be able to provide the first-class measurement currently. The dynamic topography may be used to distinguish the dynamic behavior of normal corneas from that of keratoconus.

**Electronic supplementary material:**

The online version of this article (doi:10.1186/s12938-015-0036-2) contains supplementary material, which is available to authorized users.

## Background

The fully dynamic analysis of cornea is significant for evaluating corneal biomechanical properties [1]. Many corneal properties including the intraocular pressure (IOP), the anterior corneal curvature (ACC) and the thickness of cornea can be easily measured using some existing innovative techniques. These parameters are important for an ophthalmologist to make a decision in many clinical practices [2, 3]. However, they only provide the static or pseudo-dynamic analysis.

The optical coherence tomography (OCT) can be used to derive parameters including the anterior corneal curvature (ACC) and the thickness of different corneal layers. Another kind of techniques adopts Sheimpflug principle to create sectional images of anterior eye chamber. In commercial model Pentacam (Oculus Optikgeräte GmbH, Wetzlar, Germany), a slit-camera device swivels around the eye and generates oriented images of the anterior eye chamber rapidly. Quantitative parameters are evaluated. However, without air puff components, parameters provided by these instruments only reflect the static properties of eye. Based on the Ocular Biomechanics Modulatro (OBM), Lombardo et al. [4] tested the stress–strain curves and creep curves which were dynamic parameters. But these experiments were ex vivo and destructive. The Ocular Response Analyzer (ORA, Reichert Technologies, New York, USA) integrates the air puff component to reveal the corneal dynamic properties in vivo [5]. It generates a dynamic bi-directional applanation process for the cornea. Two independent pressure values are derived in the inward and outward applanation events. Their difference defined as corneal hysteresis is useful in the diagnosis of eye diseases [6]. It can also monitor the corneal curvatures throughout the whole deformation process. Recently, a state-of-art instrument attempts to integrate the air puff function into the spectral optical coherence tomography (sOCT) system [7, 8]. However, due to the technique limitation, both the commercial ORA and the new sOCT can only obtain the dynamic corneal parameters at a certain point.

Utilizing the high-speed Sheimpflug-camera over 4300 frames per second, Oculus launched the new model Corvis ST (Oculus Optikgeräte GmbH, Wetzlar, Germany) to integrate the air-puff function and induce the corneal displacement [9]. In each measure, the cornea moves inward until reaching a point of the highest concavity and then rebounds to its normal convex curvature. Meanwhile, the CCD records the whole process of cornea deformation including before, during and after deformation. Despite the sacrifice of the ability to generate the three-dimensional volume data in the Pentacam, it allows tracking the two-dimensional corneal response within the width of 8.5 mm during the whole deformation process [10]. The Corvis ST can also evaluate some static and pseudo-dynamic parameters including deformation amplitude, applanation length, peak distance, etc. which potentially help the screening in keratoconus [11]. Bak-Nielsen et al. [12] verified the repeatability and reproducibility of these parameters. However, the Corvis ST does not provide the intact variation of ACC during the deformation process.

Recent studies indicate the significance of the measurement of the ACC in refractive surgery [13, 14]. The ACC also considers important for the evaluation of corneal stiffness and intraocular pressure [15, 16]. Besides, ACC has the value to differentiate the cornea diseases such as keratoconus [17]. Due to the importance of ACC and the lack of fully dynamic analysis, we proposed the dynamic curvature topography based on the Corvis ST. The new parameter combines the ACC with the corresponding frame of original image to show reactions of cornea to the air puff in the whole process. The merit of our study is to form the dynamic topography for the first time which efficiently and accurately visualizes the intact variation of ACC. It may be helpful and intuitive to understand the intraocular pressure (IOP), corneal viscoelasticity and stiffness.

Since the difficulty of the task, the proposed method includes three main steps: cornea segmentation, curvature estimation and integrated visualization.

Difficulties of cornea segmentation on Corvis ST images mainly exist in the automatic detection of cornea region and the elimination of eyelash interferences. To solve similar problems for OCT images, some research groups reported their efforts by utilizing graph theory and dynamic programming framework [18]. These algorithms succeeded in automatic segmentation and corneal layer boundaries, and were robust against artifacts and low-SNR regions. But the corneal regions were only corrupted by central saturation artifacts at the corneal apex and horizontal line artifacts which are often seen in clinical OCT images. Artifacts resulting from impurities such as eyelashes and tears within Corvis ST images are distributed unevenly outside the cornea. Since the distinct distribution characteristics of artifacts, these methods are not completely applicable. Koprowski et al. [19] took the threshold setting method and well-known Canny edge detection method to determine the outer limit of the cornea. They worked well when the images contained distortion and speckle noise only. In our study, artifacts like eyelashes and tears were further considered to improve the cornea detection. Due to the contrast invariant identification of the structure of objects under varying lighting conditions and uneven tissue reflectivity, the phase symmetry and asymmetry theory introduced by Kovesi [20] have been successfully used in the segmentation of many medical images. In our method, the phase symmetry, asymmetry are utilized with the prior knowledge of the corneal center, corneal boundaries and artifacts to remove disturbances and enhance segmentation accuracy. The segmentation method is conceptually simple and easy to achieve, yet capable of handling common practical images captured with unexpected noise.

After cornea segmentation, a robust algorithm for curvature estimation is applied. In previous studies, most algorithms realize the curvature estimation based on the nonlinear least squares approximation [21–23]. The Landau-new method proposed by Thomas and Chan [22] is most widely applied. The implement of the method is fairly simple to reduce the computing time and does not need to provide initial values, which is not convenient and appropriate in the whole deformation process. We therefore apply the Landau-new method to calculate values of ACC.

Integrated visualization can facilitate the interpretation of derived parameters with original images. It is critical to display the dynamic topographic map of curvature. Current ophthalmic devices present their quantitative parameters separately from the original images captured by imaging system, such as the corneal topography in the Pentacam. It may increase the difficulty in locating the concerned point. Li et al. [24] proposed the color-appearance-model based fusion method. The method fuses gray and pseudo-color images without blurring details, fading color or artifact contours. For the first time, we adapt it to combine the gamut of curvature values with original frames instead of fusing two images. The anterior corneal surface and the ACC values are fused within the appropriate width which reduces the computational cost. The aesthetic fusion images present the additional information without spoiling original images.

The remaining sections are organized as follows. “Methods” details the realization of the dynamic topographic map for anterior corneal surface. The algorithms of boundary segmentation, curvature estimation and integrated visualization are elaborated subsequently. “Experiments and results” demonstrates the validation of the proposed algorithms. Finally, issues concerned in the proposed dynamic topographic map are discussed with in “Discussion”.

## Methods

### Boundary segmentation algorithm

There are many classic traditional segmentation methods, such as the first and second order differential based operators, the Canny edge detector [25], etc. However, they are inaccurate and sensitive to noise. The energy-minimizing curves require an initialization curve close to the real contour and a lot of time for optimizing solution [26, 27]. A method for segmenting the corneal boundaries is developed in this section. The new method makes clever combination phase-based images with a priori knowledge about the position relationships between cornea and interferences. The segmentation method includes three main steps: removing artifacts, generating phase-based images and detecting the upper and lower boundaries. We will describe the steps in detail as follows.

#### Removing artifacts

**Figure 1:**
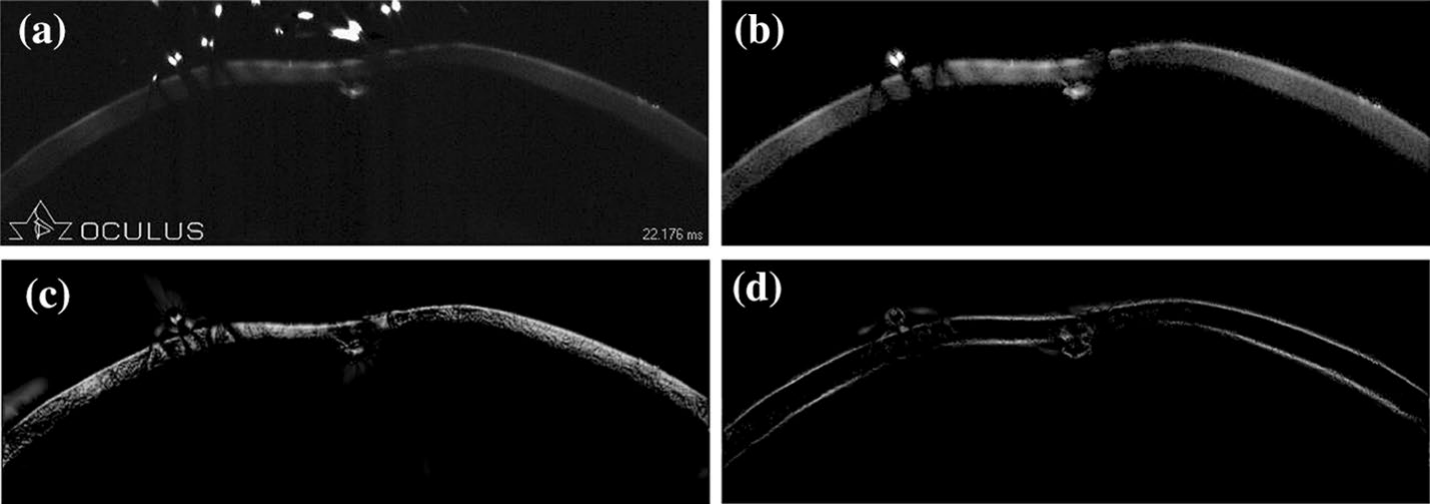
The result of preprocessing. The width of the image is 8.5 mm. And the image is one frame of the Corvis ST video with resolution of 200 × 576 pixels. **a** The original corneal Corvis ST image, **b** the gray image after artifacts removal, **c** phase symmetry-based image, **d** phase asymmetry-based image.

Sheimpflug principle allows rapid imaging for the anterior eye chamber. However, the obtained images may suffer from problems of different impurities, low contrast and uneven illumination which increase the difficulty in detecting cornea surfaces. While many methods segment the high-quality corneal images, they fail to segment images corrupted by artifacts accurately. Effective removal of impurities is therefore very important. In Corvis ST images, the size of impurities is much smaller than the cornea. We adopt this as a prior knowledge to remove the impurities. Specific steps are as follows. The cornea gaps caused by uneven illumination are firstly connected through morphological closing method with a linearly structural element (sized 10 × 60 pixels). The area of different objects, such as the impurities and cornea, is then calculated. A binary image which only contains the cornea is finally generated by setting a threshold of area. Since the area of each impurity is smaller than that of cornea obviously, the selection of the threshold is not so strict. In this paper, the value is the 1/3 size of the biggest area. Figure 1 shows the results after removing the artifacts. A little noise of eyelashes remains in Figure 1b because of its connection to the cornea. We will eliminate its impact in “Detecting the upper and lower boundaries”.

#### Generating the phase-based images

Kovesi [20] proposed a new low-level feature, named the symmetry and asymmetry of the local phase. It requires no prior position of the object and is robust to noise. We briefly review the theory. Any discrete signal can be represented by sine and cosine functions with specific amplitudes. At the points of symmetry, frequency components are at either the peak or valley points in their cycles. In contrast, at the points of asymmetry, all frequency components are at their inflection points. In some sense, phase symmetry represents a generalization of delta features and phase asymmetry represents a generalization of step edges. A series of log-Gabor filters are utilized to extract the local phase information. The phase symmetry is given by

1 $$ Sym(x) = \frac{{\sum\nolimits_{n} {\left\lfloor {[\left| {e_{n} (x)} \right| - \left| {o_{n} (x)} \right|] - T} \right\rfloor } }}{{\sum\nolimits_{n} {A_{n} (x) + \varepsilon } }} $$

where *e*_*n*_(*x*), *o*_*n*_(*x*) and *A*_*n*_(*x*) are the even symmetric part, the odd symmetric part and the magnitude response of point *x* at the scale *n*, respectively. The factor *T* is a scale specific noise compensation term. The term *ɛ* is a small positive constant to avoid division by zero. At the point of symmetry, the magnitude of the even-symmetric filter output is larger than that of odd-symmetric filter.

At points of highly asymmetry, the absolute values of the even and odd-symmetry filter outputs are reversed from symmetric case. Thus, a measure of asymmetry can be expressed as

2 $$ ASym(x) = \frac{{\sum\nolimits_{n} {\left\lfloor {\left[ {\left| {o_{n} (x)} \right| - \left| {e_{n} (x)} \right|} \right] - T} \right\rfloor } }}{{\sum\nolimits_{n} {A_{n} (x) + \varepsilon } }} $$

The extension to 2D local analysis is performed by applying the 1D analysis in multiple orientations and forming a weighted sum of the results. The Corvis ST image is then convolved with oriented filter banks to calculate the phase symmetry and asymmetry in 2D. Figure 1c, d display the results of applying phase symmetry and asymmetry approach after image preprocessing. The background pixel values of images are very low, which demonstrates the efficiency of the phase symmetry and asymmetry approaches in detecting delta and edge features in images.

#### Detecting the upper and lower boundaries

A priori knowledge should be applied to effectively distinguish the upper from lower boundaries at the same time. Owing to the position of boundaries, the contour detection is realized by first obtaining the center line which divides the cornea into upper and lower parts. Points on the two boundaries are then identified and applied to fit the curves. These two steps are detailed as follows.

The phase symmetry-based image is denoted as image *I*_*center*_ for convenience. After setting a threshold of gray level (the gray value of black and bright points are of great differences in the range of 0–1, so setting the value of threshold is not so strict. In this paper, we set it as 0.1), the bright points whose gray values are bigger than the threshold are obtained. The x-coordinates of these points located in the column *j* of image *I*_*center*_ are stored in the array *L*_*j*_. Here, the coordinates (*x*, *y*) and the intensity *m*_(*x*, *y*)_ of points on the central curve can be expressed as:

3 $$ (x,y) = (median(L_{j} ),j),\quad j = 1, \, 2, \ldots ,N, $$

4 $$ m_{(x,y)} = I_{{(median(L_{J} ),j)}} $$

where *N* is the number of columns in the image *I*_*center*_. The *x* and *m* are the median value of the array *L*_*j*_ and the intensity of midpoint obtained in each column *j*.

To reduce the interference of remaining artifacts, the fifth-order polynomial curve fitting method is adopted for the central line estimation instead of the linear interpolation method.

Similarly, in order to get the upper and lower boundaries, phase asymmetry-based image is used for the detection. According to the position relationship between the center line and the boundaries, there are two bright points closest to the central line in each column. The one above the central line belongs to the upper boundary, and the other one belongs to the lower boundary. Since the impurities are at the periphery of the cornea, points on the contours obtained by the proposed method are closer to reality.

**Figure 2:**
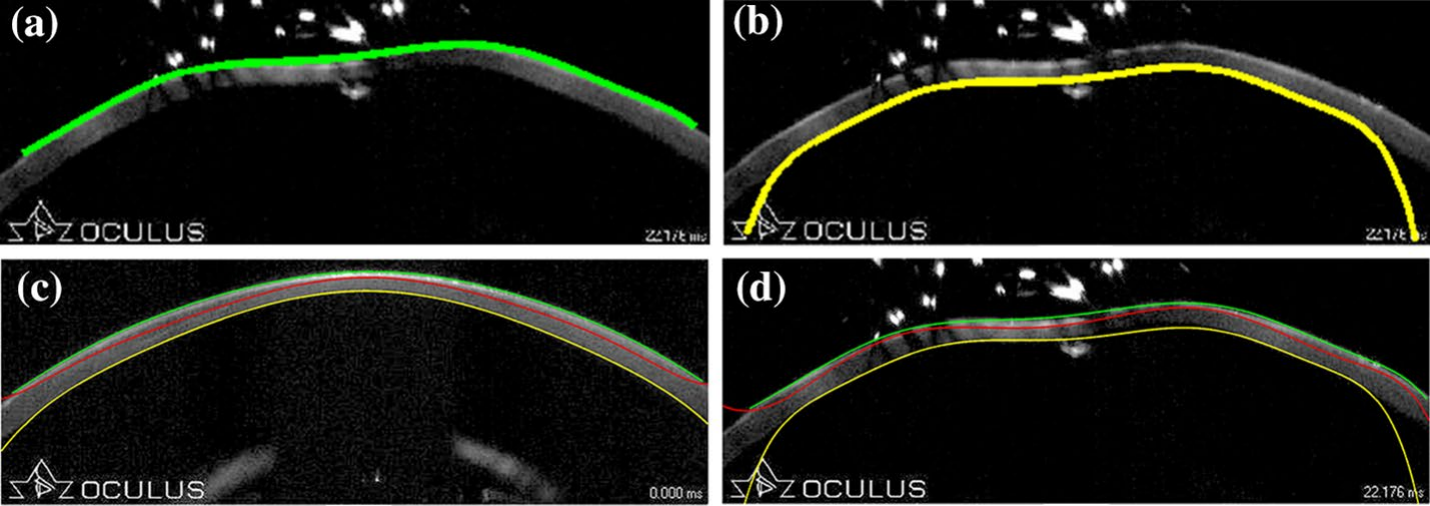
Results of boundary detection. The upper and lower boundary are represented in *green* and *yellow*, while the *red line* reveals the central line. **a** The detection of upper boundary, **b** the detection of lower boundary, **c** the segmentation result of image with little impurity, **d** the segmentation result of image with many impurities.

Figure 2 shows the results of detection. The upper and lower boundaries are represented in green and yellow, while the red line reveals the central line. Figure 2a, b are the results of upper and lower boundary detection, respectively. Because of the uneven illumination, the phase symmetry and asymmetry points are not obvious and their values are almost zero in left and right sides. Some of the curves are therefore not very accurate at both ends. The inaccurate segmentation in the end parts however does not affect the overall results. Because only the segmentation of corneal deformation part (central area, which mostly takes up 2/3 length of the whole image) matters.

### Curvature estimation

Points on the anterior corneal surface are discrete and small fluctuations may produce big error. Thus, it becomes inaccurate to calculate the curvature directly through the curve expression. Thomas proposed a simple approach for the estimation of circular arc center and its radius [22]. By considering the errors in area rather than a length, the method is implemented more easily than the iterative algorithms with reduced computing time. The approach does not need initial values of the locations of the center and radius of circular arc. So it is convenient and appropriate when dealing with Corvis ST sequential image. Besides, Thomas verified the method through changing noise factor and arc length. Due to these merits and applicability, the method is applied to calculate the radius of curvature with a set of coordinates which we have already gotten.

We briefly review the general idea behind the theory in the context of radius of curvature estimation. On the upper boundary, we take 27 pixels long of coordinates (*x*_*up*_, *y*) on each side of the targeted point successively and get a set of coordinates (*x*_1_, *y*_1_) … (*x*_i_, *y*_*i*_) … (*x*_*N*_, *y*_*N*_). They are assumed to belong to the tangent circle with center (*x*_*c*_, *y*_*c*_) and radius *R*. The number of points should be neither too long nor too short for the tradeoff between the length of arc and accuracy. Summing up all the squares of errors we have

5 $$ J = \sum\limits_{i = 1}^{N} {[\pi R^{2} - \pi \{ (x_{i} - x_{c} )^{2} + (y_{i} - y_{c} )^{2} \} ]^{2} } $$

Through the differentiation of Eq. (5) with respect to *R*, *x*_*c*_, *y*_*c*_ respectively and a series of calculation and simplification, the radius of curvature is obtained.

### Curvature tomography drawing

**Figure 3:**
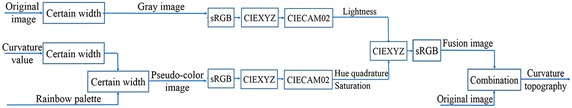
Block diagram of the fusion algorithm. These submodules are, in order, acquisition of original image and calculation of curvature of the anterior cornea, normalization of the curvature values, width extension, transformation, extraction of lightness, hue quadrature and saturation, and reverse transformation.

It is a difficult task for clinicians to mentally translate and integrate the ACC values with corneal gray images. Thus, merging the two information of cornea into a single image is very important and facilitates the diagnosis or therapy in medical practice. Fusion technique [28–33] has been proven successful to deal with the problem. Li et al. [24] first introduced a color-appearance-model for medical image fusion. Without the defects of blurred details, faded color and artifact contours, the method is proved to be superior to traditional methods including color-space-based methods, transparency technique, alternating display technique. Accordingly, we adapt this color-appearance-model based fusion method to visualize curvature information. To our knowledge, the objects of fusion are traditionally two different images. Combination parameters with images are firstly proposed and some preprocessing are needed before the fusion. First, the derived curvature values of anterior cornea are expanded to preserve its original profile. They are then visualized in pseudo-color onto the original Corvis image within the relevant regions. Figure 3 illustrates the whole process of image fusion.

Using the CIEXYZ color space as intermediary, the method transforms images from standard sRGB tristimulus values to color appearance model of International Commission on Illumination published in 2002 (CIECAM02). It then merges the hue quadrature and saturation of pseudo-color and the lightness of gray image together. The reverse transformation from CIECAM02 to sRGB is finally executed. In this framework, since we have recorded the coordinates of upper boundary points, a certain width of strip (for example, 21 pixels) centered on the upper boundary in original corneal image are extracted and viewed as the gray-scaled image. After the corresponding radius of curvature values are copied to the same width, the parameter band is normalized into a rainbow palette to get the pseudo-color image. The width can ensure the visual effect and save calculation time. After following the steps of the fusion process, the new color strip is generated. It is used to replace the original corneal image in corresponding position for display.

In order to intuitively observe the dynamic change of the anterior corneal surface in different stages, the curvature topography of the Corvis ST video is proposed. The same color bar should be used in three stages. Because the range of curvature radius value before and after deformation is smaller than the one during deformation, values are normalized nonlinearly as Eq. (6) to achieve a richer color bar.

6 $$ R\_new = \left\{ {\begin{array}{*{20}l} {0(R \le - 35) \, } \hfill \\ {0.009\cdot(R + 35)( - 35 < R \le 7) \, } \hfill \\ {0.1333\cdot(R - 7) + 0.3761(7 < R \le 10) \, } \hfill \\ {0.009\cdot(R - 10) + 0.7761(10 < R \le 35)} \hfill \\ {1(R > 35)} \hfill \\ \end{array} } \right. $$

## Experiments and results

### Materials

Forty eyes in normal group and 30 eyes in keratoconus group were included. One randomly selected eye of each person in normal group and one or two keratoconic eyes in the keratoconus group were examined. The normal and keratoconus group ranged from 19 to 45 and 16–40 years old, respectively. Patients were excluded from the study if they had any ocular pathology other than keratoconus. Data was collected from August 2012 to December 2013 at Chinese General Hospital from the People’s Liberation Army, Beijing, China (Chinese PLA General Hospital). The data set was created by selecting consecutive ten frames of each stages including before, during and after deformation. Manual segmentation is performed by two specialists from Chinese PLA General Hospital. In order to reduce the measurement error, every manual data, which would be utilized as the standard, was the mean value of twice measures of each expert without being aware of the duplication. The processes of built-in method are recorded by the CamStudio software. Then the data of built-in is obtained according to the colors information (in the recorded video, the red and green lines represent upper and lower boundaries, respectively).

Due to no ACC values in Corvis ST, Pentacam was used to evaluate our estimation of curvature before deformation. Eleven subjects (ranging from 19 to 40) were enrolled both in normal group and keratoconus group. Eleven points were then chosen from the corneal center to two ends on the curvature topographic map of each object. The total number of points was 242 (2 × 11 × 11).

### The verification of segmentation algorithm

To verify the accuracy and validity of our proposed segmentation algorithm, the results from automatic segmentation were compared with those from built-in segmentation method and the manual segmentation. We calculated the central corneal thickness (CCT) and the distance between the two surrounding “apexes” at the time when the cornea reached its highest concavity (peak distance, PD) [16] for quantitative analysis. The CCT could only show the local properties of methods, while the PD in the stage of blowing air could test the global effect.

Statistical analyses were performed with SPSS version 16.0 software (SPSS for windows, SPSS, Inc. Chicago, IL). The significances of both correlation and difference were taken into consideration. To analyze the correlation between the three methods, the Pearson’s correlation coefficient (*r*) was calculated. The *P* values of two-side T test were considered statistically significant when they were less than 0.05. In contrast, to evaluate the differences between the data of three methods, a one-way analysis of variance (ANOVA) with Tamhane’s T2 multiple comparisons was applied. The *P* values of the Tamhance test were considered no significant differences when they were higher than 0.05.

**Figure 4:**
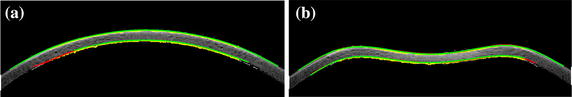
Comparisons of our (*green*), expert (*red*) and built-in (*yellow*) segmentation. In order to suppress interferences from the results of our and built-in segmentation, expert segmented the boundaries firstly. So the *red line* was shown in the bottom. **a** The comparison in the stage of before deformation, **b** the comparison in the stage of during deformation.

**Figure 5:**
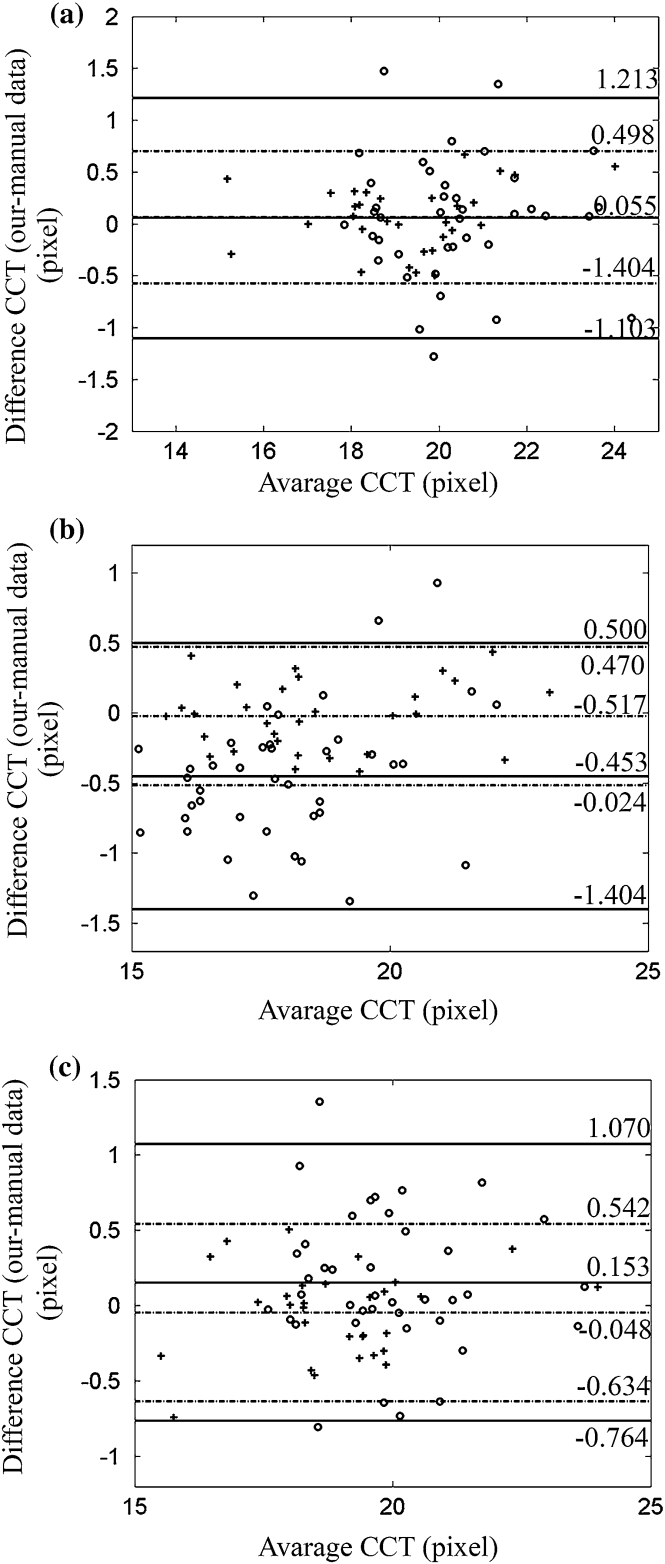
Bland–Altman plots of the CCT values. The *hollow circle* and *plus sign* represent data from normal and keratoconus group, respectively. The *solid lines* and *black dotted lines* are limits of normal and keratoconic data, respectively. **a** Our method versus manual method before deformation, **b** our method versus manual method during deformation, **c** our method versus manual method after deformation.

**Figure 6:**
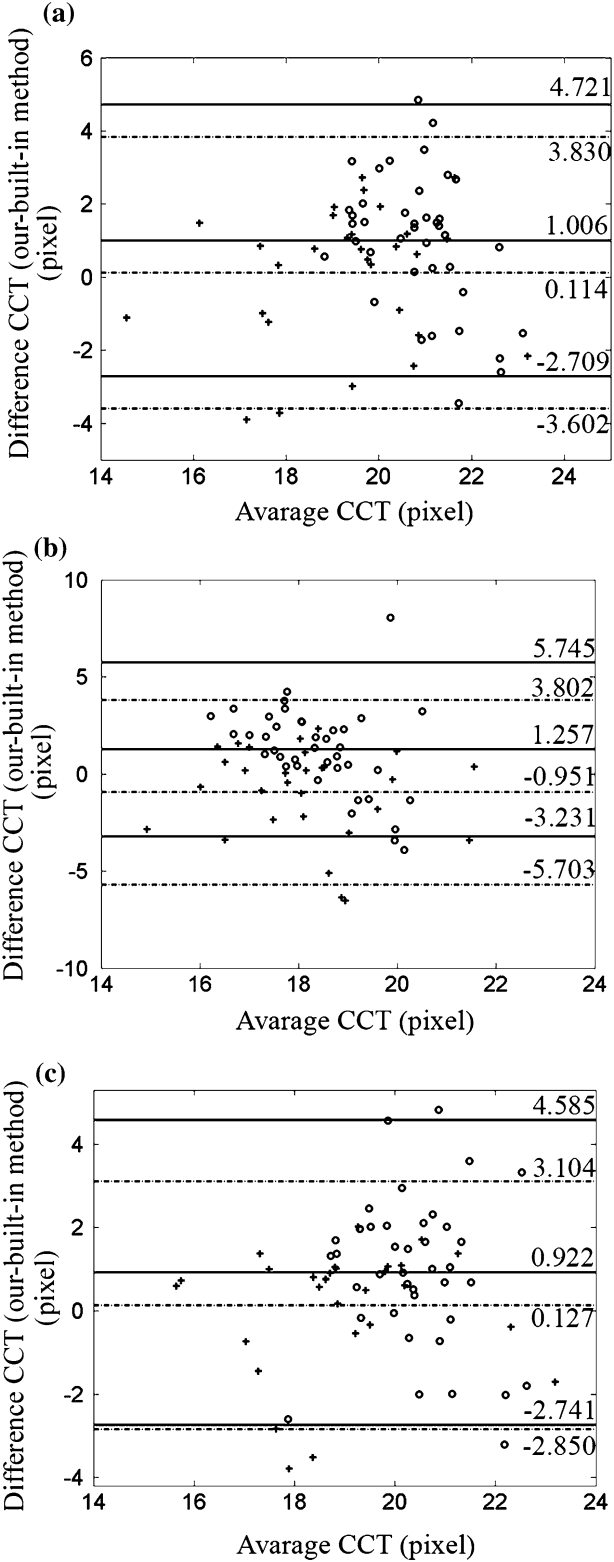
Bland–Altman plots of the CCT values. The *hollow circle* and *plus sign* represent data from normal and keratoconus group, respectively. The *solid lines* and *black dotted lines* are limits of normal and keratoconic data, respectively. **a** Our method versus built-in method before deformation, **b** our method versus built-in method during deformation, **c** our method versus built-in method after deformation.

**Figure 7:**
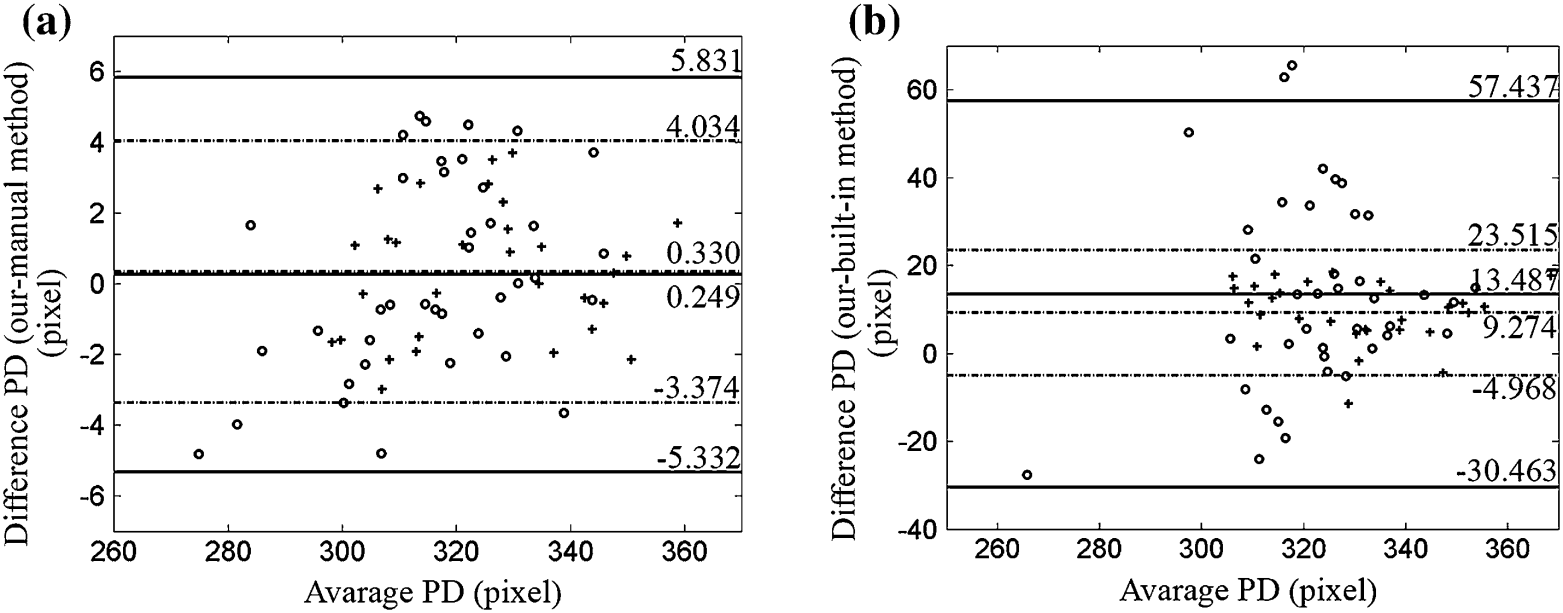
Bland–Altman plots of the PD values. The *hollow circle* and *plus sign* represent data from normal and keratoconus group, respectively. The *solid lines* and *black dotted lines* are limits of normal and keratoconic data, respectively. **a** Our method versus manual method, **b** our method versus manual method.

The qualitative results are showed in Figure 4 with our automatic segmentation (green) overlaid with manual method (red) and built-in method (yellow). The quantitative analysis was also performed. Tables 1 and 2 demonstrate the results of the comparison of CCT values in normal group and keratoconus group obtained by three methods, respectively. Table 3 reports the analysis of pair comparison of PD values in two groups between different methods. Correlation analysis shows a high correlation between our method and manual method (*P* ≤ 0.01, two-side T test). The *r* values of CCT values between our method and manual method in three stages are all close to 1, which reveal a strong positive correlation between the two methods. The *r* values of CCT between our method and built-in method are smaller than the one between our method and manual method, especially in the “during deformation” stage. Similarly, the *r* value of PD between manual method and our method is 0.9943 in normal corneas and 0.9972 in keratoconus, respectively. Besides, the differences in CCT and PD values between our method and manual method are not significant (*P* > 0.05, Tamhance test). Figures 5, 6 and 7 show the Bland–Altman plots [34]. Each pixel represents 0.016 mm. The data are expressed by hollow circle in normal group and plus sign in keratoconus group. The solid lines and black dotted lines represent the mean difference, mean difference + 2SD and mean difference − 2SD of normal and keratoconic data, respectively. The middle line represents the mean difference which estimates bias. All the middle lines are close to 0. And the maximum percentages of points outside the limit lines are 7.5 and 5% in Figures 5 and 6, respectively. The results reveal a strong agreement between our method and the other two methods. Figure 7 shows that the mean difference between our method and manual method is also close to 0 and all the points are located inside the limit lines. The mean difference of PD between our method and built-in method is 13.4866 and 9.2736 pixel in normal and keratoconus group, respectively. Meanwhile, the percentage of points outside the limit lines is 3.3%.

**Table 1:** Pairwise comparison of CCT values in different stages (normal group)

Stages	Our method versus manual method			Our method versus built-in method		
	*r*	Two-side T test	Tamhance test	*r*	Two-side T test	Tamhance test
Before deformation	0.969	0.01	0.998	0.938	0.01	0.080
During deformation	0.9835	0.01	0.580	0.654	0.389	0.627
After deformation	0.9763	0.01	0.956	0.934	0.01	0.515

**Table 2:** Pairwise comparison of CCT values in different stages (keratoconus group)

Stages	Our method versus manual method			Our method versus built-in method		
	*r*	Two-side T test	Tamhance test	*r*	Two-side T test	Tamhance test
Before deformation	0.9926	0.01	0.999	0.662	0.03	0.120
During deformation	0.9963	0.01	1	0.234	0.222	0.564
After deformation	0.9928	0.01	0.999	0.672	0.01	0.064

**Table 3:** Pairwise comparison of PD values in normal and keratoconus group

Groups	Our method versus manual method			Our method versus built-in method		
	*r*	Two-side T test	Tamhance test	*r*	Two-side T test	Tamhance test
Normal group	0.9943	0.01	1	0.326	0.043	0.971
Keratoconus group	0.9972	0.01	1	0.837	0.01	0.292

### The verification of curvature radius estimation

**Figure 8:**
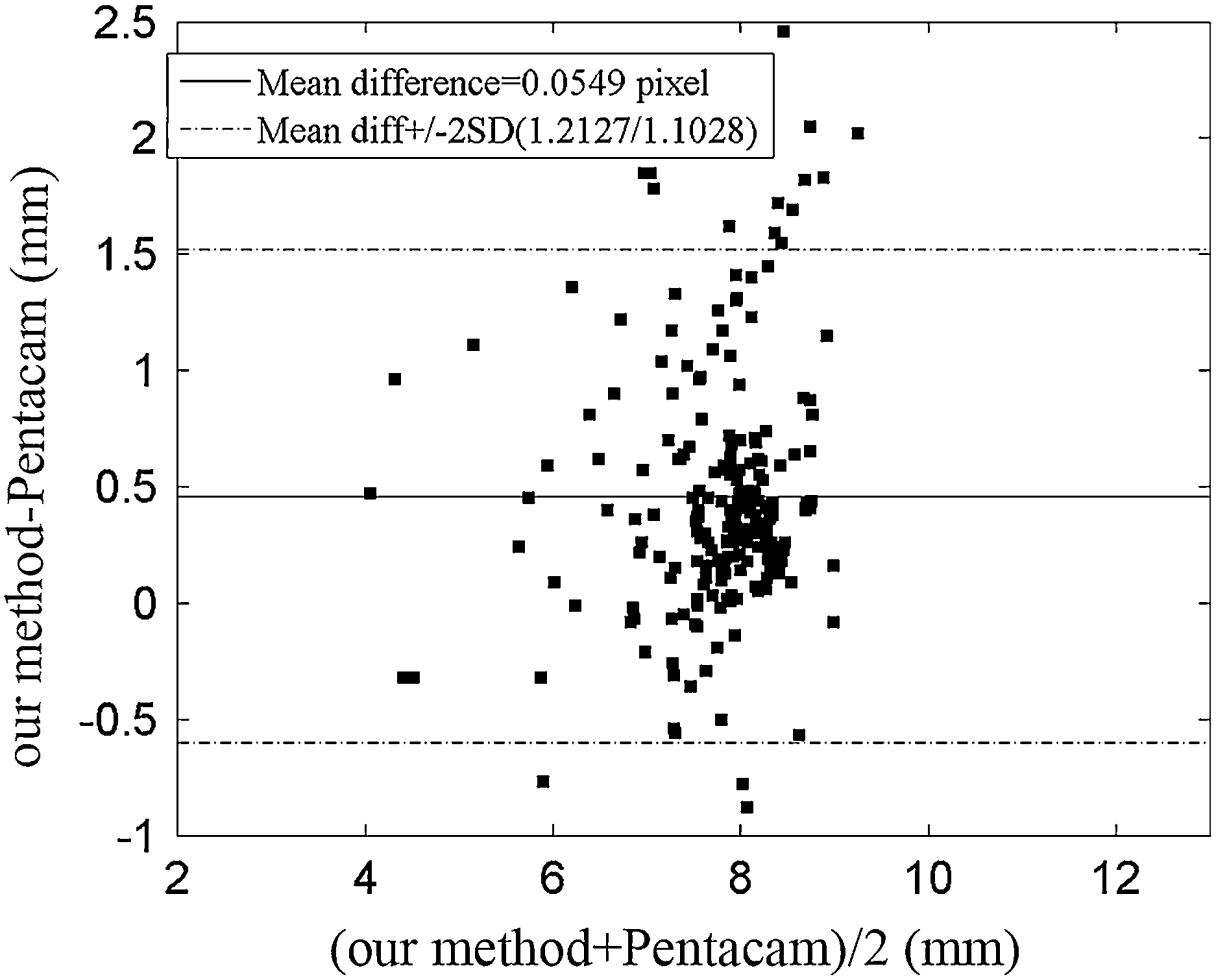
Bland–Altman plots of radius of curvature values obtained from our methods and the Pentacam. Then mean difference is close to 0. The mean difference + 2SD and mean difference − 2SD are 1.2127 and 1.01028, respectively. Besides, the percentage of points outside the limit lines is 10.4.

Up to this point, we have focused on the evaluation of the boundary estimation accuracy. With the segmentation result, we can calculate the corneal curvature radius which is actually meaningful for clinical diagnosis. The Pentacam software is used for standard clinical static cases. Thus, our estimated ACC values in the “before deformation” stage were compared with Pentacam. The accurate estimation in this stage can ensure the exactness in the other two stages to a certain extent. The standard deviation (SD) and the Pearson coefficient (*r*) compare the curvature measured by our method and Pentacam. The SD and *r* are 0.711 and 0.8925, respectively. Figure 8 shows agreement between our method and Pentacam.

### Curvature topographic map

**Figure 9:**
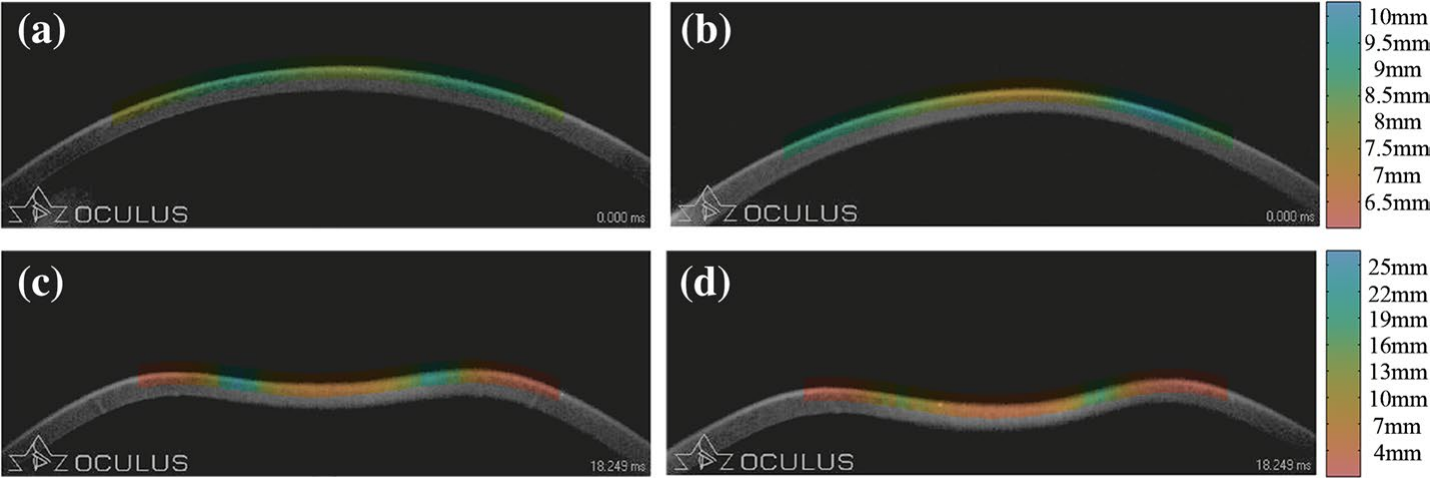
Average curvature topographic maps. **a** The map of normal corneas before deformation, **b** the map of keratoconic corneas before deformation, **c** the map of normal corneas during deformation, **d** the map of keratoconic corneas during deformation.

In order to explore the role of the curvature topographic map in observing dynamic behavior of normal and keratoconic corneas in different stages, we gave the average topographic maps for the two types of eyes based on the subjects tested in “The verification of curvature radius estimation”. Figure 9 shows the differences between normal and keratoconic corneas, which can be observed from the image. Through static curvature topographic map produced before deformation, we can see that the curvature radius of normal corneas is bigger than keratoconic corneas in the central place and changes more smoothly from the center to two sides. The curvature radius also changes more sharply in the center and more moderately in the remote place than the keratoconus.

As we mentioned before, Corvis ST records the total corneal response. Hence, we can apply the dynamic curvature topographic map to analyze the dynamic behavior of corneas.

From the Media 1 (see Additional file 1), we can easily see the differences of corneal responses in the whole process. The dynamic curvature topography map of normal cornea shows a faster reaction and a larger response scope to the air puff of normal eyes. After the air puff is removed, the normal cornea restores to its original state more quickly, which have not been proposed in previous studies.

## Discussion

It is common and inevitable that there are many impurities in Corvis ST images, especially in the “during deformation” stage. While the built-in method failed to deal with these kinds of images, our method still succeeded. It revealed that our segmentation method is more robust to impurities than built-in method. The Bland–Altman and Pearson’s correlation coefficient showed a strong positive correlation between our method and manual method. However, the *r* values of CCT between our method and built-in method were not big in the “during deformation” stage. The Corvis ST recorded the dynamic process of cornea responding to the air puff. The corneal motion and shake could blur the images, which made it hard for Corvis ST to segment the corneal boundaries, particularly the lower boundary. That may explain why the correlation between our method and built-in method is not strong. Besides, the difference between our method and the built-in method of PD is obviously highly than the difference between our method and manual method. However, the measurement repeatability of PD in the Corvis ST has been reported as poor from the literatures with ICC of 0.22 [35] and 0.52 [36]. This maybe the reason why there is no sufficient agreement between the PD values from our method and built-in method. Taken above analysis, the proposed automatic segmentation algorithm can be a proper alternative method in cornea segmentation.

The Person coefficient between the results of our method and Pentacam is insignificant mainly for two reasons. Firstly, Corvis ST and Pentacam software are two different equipments. Their imaging proportion and range are different. The central points are therefore not completely consistent. Second, the Pentacam provides the parameters of total 360° cornea in static state, while the Corvis ST gives parameters of the corneal central line changing over time. If eyes are moving, there may be a deviation of the central line between Pentacam and Corvis ST. However, the two data sets should be consistent to a certain extent because those two methods examined the same subjects in roughly the same positions. The *r* value of 0.8925 shows a strong consistency between the two methods and then the accuracy of the Landau-new method.

From the intuitive observation of the whole process, the response to the air puff of normal and keratoconic corneas can be noticed easily. The dynamic change of corneal curvature can also make up for the defect of other medical instruments such as the Pentacam, which only provides static tomography and limits the research of corneal dynamic behaviors. The intuitionally whole deformation may be helpful to analyze the corneal biomechanical properties which is the hot point of current research.

## Conclusion

In this paper, we presented the dynamic curvature topography for the anterior corneal surface variation measurement in human eyes based on Corvis ST. Our contributions are threefold. First, a phase-based automatic segmentation method is proposed. The method utilizes prior knowledge to remove impurities and trace boundaries. Second, we fuse reasonable range of corneal image with nonlinear normalized curvature radius values. The most important is that the dynamic curvature tomographic map is proposed for an intuitive way to observe the dynamic change of the anterior corneal surface in the whole air stream process. The experiment results show that the proposed segmentation method has better robustness and higher accuracy than built-in method. The correctness of curvature radius was also presented by the close agreement with that of Pentacam.

However, the dynamic curvature radius change through visual effects should be quantitatively analyzed. Besides, we will look at the potential for applying the dynamic topographic map to classify different corneal pathologies in the future work.

## Additional files

Additional file 1: Media 1. The video of corneal responses to the air. The above section shows the reaction of normal cornea. The following section shows the reaction of keratoconus.

## Abbreviations

OCT: optical coherence tomography
ACC: anterior corneal curvature
ORA: ocular response analyzer
sOCT: spectral optical coherence tomography
IOP: intraocular pressure
CIECAM02: color appearance model of International Commission on Illumination published in 2002
CCT: central corneal thickness
PD: peak distance
ANOVA: a one-way analysis of variance
SD: standard deviation.
